# A Novel Frequency Selectivity Approach Based on Travelling Wave Propagation in Mechanoluminescence Basilar Membrane for Artificial Cochlea

**DOI:** 10.1038/s41598-018-30633-0

**Published:** 2018-08-13

**Authors:** Yooil Kim, Ji-Sik Kim, Gi-Woo Kim

**Affiliations:** 1 Department of Mechanical Engineering, Inha University, Incheon, 22212 South Korea; 2 Department of Naval Architecture and Ocean Engineering, Inha University, Incheon, 22212 South Korea; 3 School of Nano & Adv. Mater. Engineering, Kyungpook National University, Sangju, 37224 South Korea

## Abstract

This study presents the initial assessment for a new approach to frequency selectivity aimed at mimicking the function of the basilar membrane within the human cochlea. The term cochlea tonotopy refers to the passive frequency selectivity and a transformation from the acoustic wave into a frequency signal assisted by the hair cells in the organ of Corti. While high-frequency sound waves vibrate near the base of the cochlea (near the oval windows), low-frequency waves vibrate near the apex (at the maximum distance from the base), which suggests the existence of continuous frequency selectivity. Over the past few decades, frequency selectivity using artificial membranes has been utilized in acoustic transducers by mimicking cochlea tonotopy using cantilever-beam arrays with defined physical parameters such as length and thickness. Unlike the conventional cantilever-beam array type, the travelling wave propagation based-mechanoluminescence (ML) membrane made of ZnS:Cu- polydimethylsiloxane (ZnS:Cu-PDMS) composite that we describe here provides new frequency selectivity more similar to that demonstrated by the human membrane. Here, we explored the potential of the ML membrane to deliver new frequency selectivity by using a non-contact image sensor to measure visualized frequencies. We report that the ML basilar membrane can provide effective visualization of the distribution of strain rate associated with the position of maximal amplitude of the travelling wave.

## Introduction

Since hearing loss is mainly attributed to the loss of hair cells in the cochlea, one of the most effective medical treatments for hearing loss is a cochlear implant (CI)^1^. However, there are several known problems with conventional CIs, including their high price and incompatibility with extracorporeal devices for calculating fast Fourier transform (FFT). To overcome the limitations of the conventional CIs, artificial basilar membranes (ABMs) have been developed over the past few decades. Ever since Georg von Békésy was awarded the Nobel Prize in Physiology or Medicine for his research on the function of the cochlea in the mammalian hearing organ^2,3^, extensive research efforts have been focused on the role of the cochlea including basilar membrane and its ability to exhibit frequency selectivity.

**Figure 1:**
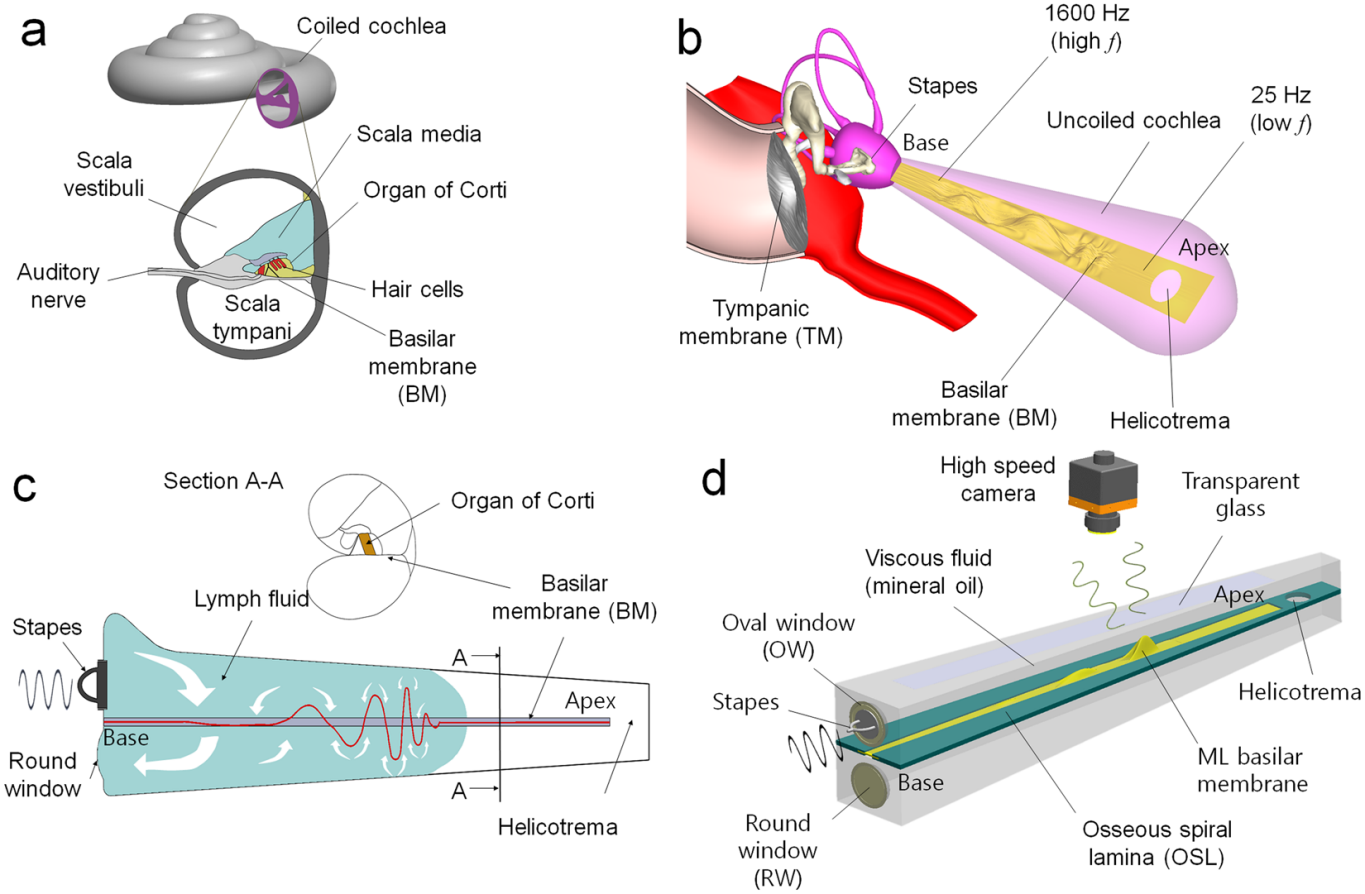
Traveling wave-based frequency selectivity approach in ML basilar membrane. (**a**) Anatomy of human auditory system; (**b**) the uncoiled cochlea showing frequency selectivity to different regions of the basilar membrane; (**c**) the travelling motion of the basilar membrane (BM) in response to harmonic excitation; (**d**) the schematic of an artificial cochlea based on the ML basilar membrane.

The cochlea, an important auditory component of the inner ear, is a spiral-shaped cavity in the bony labyrinth, making 2.5 turns around its axis in humans (Fig. 1(a)). The human basilar membrane (BM) within the cochlea of the inner ear is a resonant structure that varies in width and stiffness and appears as a long trapezoidal vibrating structure (Fig. 1(b)). The cochlea itself is a spiral, hollow, conical chamber of bone, which is connected to the oval window (OW), to the apex (the top or center of the spiral). A core component of the cochlea is the basilar membrane separating two chambers in the coiled tapered tube of the cochlea. The BM is the principal structural element because it determines the cochlea dynamics. Models of cochlear mechanics are initially attempted by Helmholtz in 1877 to explore perception of sound tones, and followed by Gold *et al*.^4^ to interpret the sharp tuning observed in the cochlea. Many different types of cochlear model have been proposed by including various physical models^5–9^. More recent models have been used to demonstrate that a cochlear amplifier mechanism is necessary to explain the sharply tuned response of the BM to single tone stimulation^10^. Most recent cochlea models are developed based on the travelling wave propagation theory suggested by Békésy, and nicely summarized in^11^. The uncoiled human cochlea, a fluid (endolymph)-filled long tubular structure, was generally used for cochlea model because neither the coiling nor uncoiling are believed to play a major role in the mechanics of the cochlea^12^. The cochlear structures include three scalae (or chambers): the scala vestibuli (SV), the scala tympani (ST) terminating at the round window (RW), and the scala media. The helicotrema is a connecting site where the SV and the ST merge at the apex of the cochlea, as shown in Fig. 1(c). Cochlea dynamics can then be analyzed in terms of two fluid chambers separated by the BM. The BM exhibits transverse isotropic property, which implies that the BM is stiffer in the transverse direction than longitudinally because of embedded transverse collagen fibers^13–15^. However, recent researches on gerbil BM indicate that this orthotropy may not be particularly significant^16,17^. Therefore, most cochlea models neglect the longitudinal stiffness of the BM^18^.

Different regions of the BM in the organ of Corti, the sound-sensitive portion of the cochlea, vibrate at different sinusoidal frequencies due to variations in thickness and width along the length of the membrane, causing a travelling wave propagation (Fig. 1(c)). The vertical vibrations result in the deflection of the stereocilia bundles of outer hair cells in the organ of Corti. This event opens the ionic channels leading to an action potential and stimulates the auditory neurons connected to the brain. The cochlea, thus, forms a highly sensitive multi-channel frequency filter capable of decomposing incoming sound signals into approximately 3,500 channels (bands) equivalent to the number of inner hair cells, which is called frequency selectivity. Therefore, ABMs are fundamentally acoustic sensor capable of mimicking the function of the basilar membrane within human cochlea (i.e., frequency selectivity)^19^. White and Grosh^20,21^ fabricated micro-machined, fluid-filled, variable-impedance waveguides, intended to mimic the mechanics of passive mammalian cochlea, and experimentally examined their frequency selectivity. However, most advances concerning passive mechanical frequency selectivity have been achieved through use of MEMS-beam-array-based ABMs with varying geometric parameters, such as beam length^22,23^, beam width^24,25^, and beam thickness^26^. In addition, the output of cantilever beam array-based ABMs was measured by using various acoustic-to-electric transducers, such as the piezoelectric^22–26^ and triboelectric^27^ transducers, for self-powered devices. Although most previously proposed biomimetic ABMs comprising finite number of cantilever beam array (or comb-shaped) structures have demonstrated promising results, they suffer from relatively low-resolution owing to their number of channels being much less compared to the 3,500 channels of the human auditory system^28^.

In this study, the travelling wave propagation-based mechanoluminescence (ML) basilar membrane was explored for new passive frequency selectivity principle, and it was implemented by means of an initial prototype design comprising an artificial membrane made of a thin ML composite (Fig. 1(d)). When compared against conventional cantilever-beam-array-based approaches, the proposed approach demonstrated more similarity with the fluid–structure interactive actual frequency selectivity of the human cochlea. The term ML describes the phenomenon of visible light emission from phosphors (inorganic host and impurity metal activator particles) in response to mechanical stimuli, such as friction, tension, fracture, and compression. Recently, this fascinating ML material has enabled us to explore various advanced sensor applications^29–31^. Working principle of the proposed frequency selectivity approach could be described as follows: the visible light emission from the ML membrane induced by the large deformation (i.e., strain rate) caused by traveling wave propagation is measured directly using a noncontact image sensor, and is converted to the position of peaked deformation that is proportional to the frequency of the input wave (or sound). Compact image sensors are, therefore, only required to detect the position of peak deformation, and act as inner hair cells within the human auditory systems. While the resolution of conventional ABMs, including human BM, depends on the finite number of cantilever beams or hair cells, the number of channels of the proposed frequency selectivity method theoretically becomes infinite (i.e., continuous) owing to the use of non-contact image sensors to measure visualized frequencies on the ML membrane.

## Fluid-structure Interaction Acoustic Analysis

**Figure 2:**
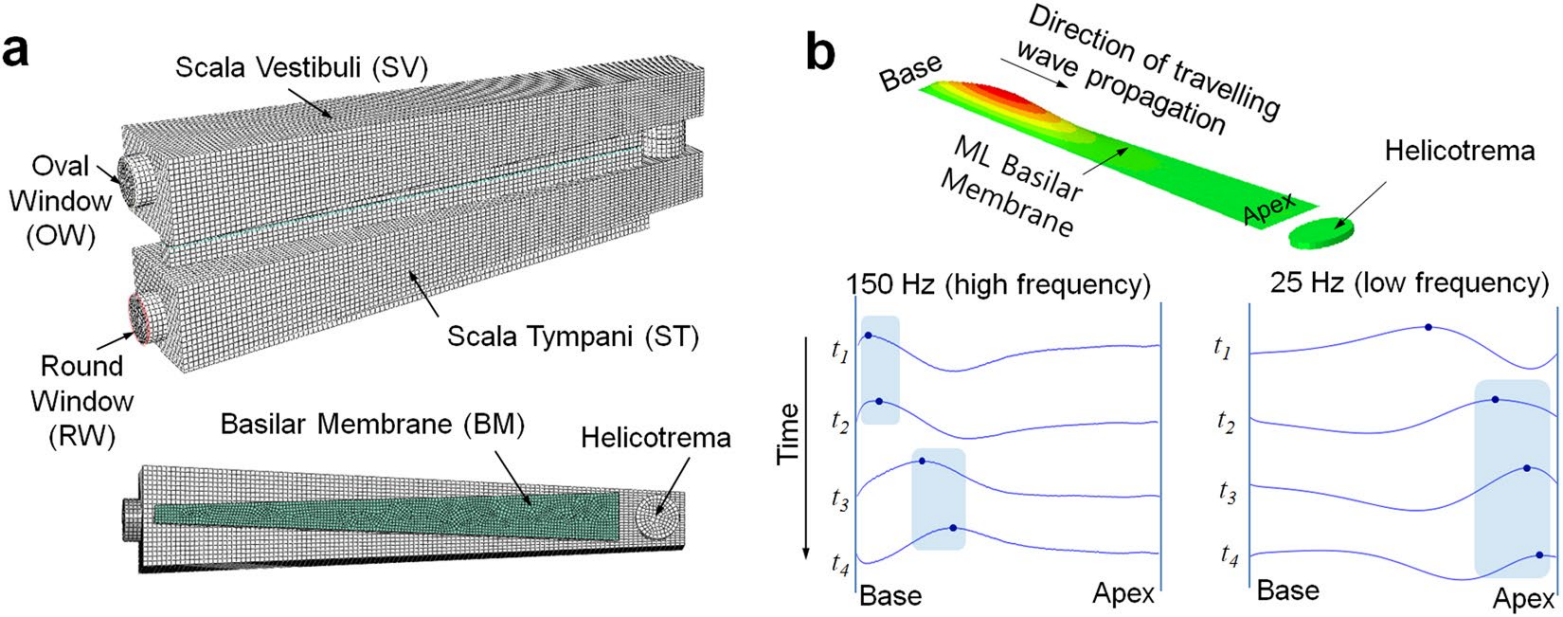
Fluid-structure interaction acoustic analysis. (**a**) Finite element model (mesh) for the prototype artificial cochlea; (**b**) travelling wave propagation patterns for different frequencies, indicating frequency selectivity.

**Figure 3:**
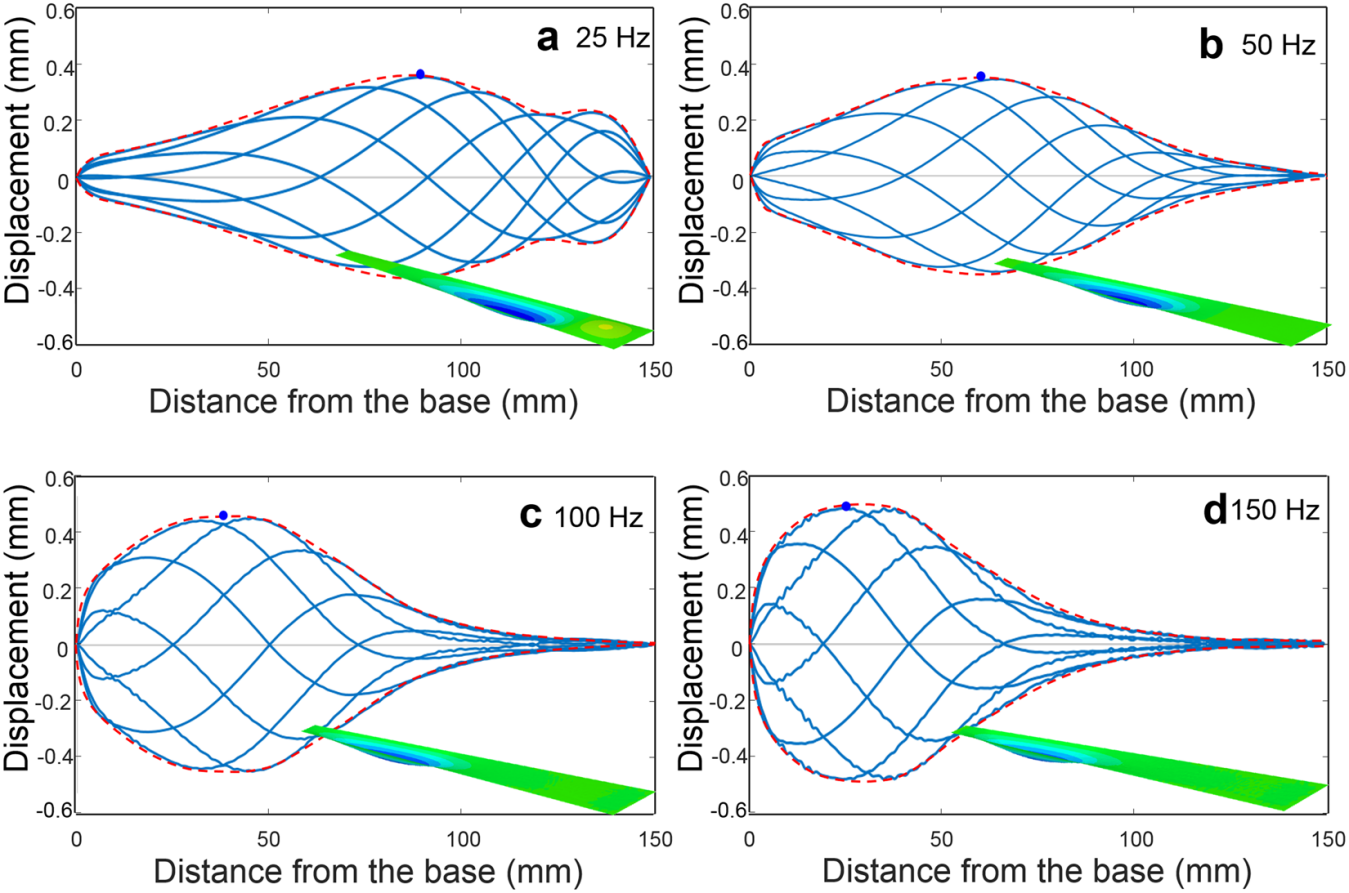
Time course of ML basilar membrane vibrations (displacement) at different frequencies. (**a**) 25 Hz; (**b**) 50 Hz; (**c**) 100 Hz; (**d**) 150 Hz; : envelopes, : the position where the vibrating ML membrane exhibits the maximum amplitude; inset: vibrating ML basilar membrane, blue contours represent the area where the vibrating ML membrane exhibits the maximum amplitude (i.e., strain rate), inset: Finite element model view.

The fluid structure interaction acoustic analysis of vibrating BM within the cochlea of the inner ear was performed first to confirm the actual travelling wave propagation motion in the ML membrane, as shown in Figure S1^32^. The relationship between an incoming frequency and the position at which a vibrating ML membrane exhibits maximum amplitude was found to be in good agreement with that reported in previous studies performed on the human cochlea^33^. Subsequently, the analysis model was modified for the initial prototype by changing the size and material properties. The configuration of the finite element (FE) model of the initial prototype, including BM, is shown in Fig. 2(a). The FE model consists of the SV, ST, BM, OW, and RW. The SV and ST were modeled by acoustic elements; the other components were modeled by shell elements. The interaction between shell elements and acoustic elements was defined using a surface-based tie constraint. A uniformly distributed dynamic unit displacement was assigned over the OW, and the nodes along the perimeter of the RW were fixed. Details of isotropic material properties of artificial cochlea prototype and fluid are summarized in Table 1. The Rayleigh damping model ($C=\alpha m+\beta k$) was used to incorporate damping responses. The ML basilar membrane is modeled using an isotropic material with a Young’s modulus of 2.2 MPa^34^. The width of the BM is increased linearly, from 6 mm (base) to 15 mm (apex), such that its shape becomes trapezoidal (measuring 150 mm in length). A petroleum-based mineral oil ($\mu =0.03\,Pa\,\cdot \,s$ at 40 °C) is used for viscous fluid (endolymph in human cochlea). A commercial finite-element analysis (FEA) code, ABAQUS, was used for all calculation in this study. A steady-state dynamic analysis was used to calculate the steady-state dynamic linearized response of a system to harmonic excitation (i.e., steady-state amplitude and phase in the form of a complex number). The positions of the peak are moved towards the base of the ML BM, as the excitation frequency increases, as shown in Fig. 2(b). The travelling wave was propagated from the base to the apex, and the position where the traveling wave reached its maximum amplitude was apparently shifted from the apex to base as the stimulating frequency increased. Figure 3 shows the time course of the vibrating BM at 25 Hz, 50 Hz, 100 Hz, and 150 Hz, respectively. A travelling wave was generated in the ML basilar membrane by harmonically exciting the OW connected with stapes. While the shape of the travelling wave envelope is skewed adjacent to the base at 150 Hz (high frequency), it is slightly skewed adjacent to the apex at 25 Hz (low frequency). However, the shape of the travelling wave envelope for low frequency showed no notable change. This observation confirmed the velocity of the ML basilar membrane in response to a harmonic velocity input of OW, for different frequencies depicted in Figure S2. From these observations based on simulation using an FE model, it is obvious that the prototype artificial cochlea using ML basilar membrane exhibits a frequency selectivity similar to that exhibited by the human cochlea except for frequency ranges (20~20 kHz (human) vs. 10~150 Hz).

**Table 1:** Isotropic material properties of prototype cochlea with ML basilar membrane.

Component	Material	Young’s Modulus (MPa)	Poisson’s Ratio	Density (kg/m^3^)	etc
BM	ML + PDMS	2.2	0.28	1,350	t = 0.6 mm
OW (rubber)	Rubber	18	0.49	950	t = 0.3 mm
RW (Latex)	Latex	1.8	0.49	920	t = 0.1 mm
Viscous Fluid	Mineral oil	2,000 (Bulk modulus)	N/A	1,062

## Traveling Wave-based Frequency Selectivity in ML Basilar Membrane

**Figure 4:**
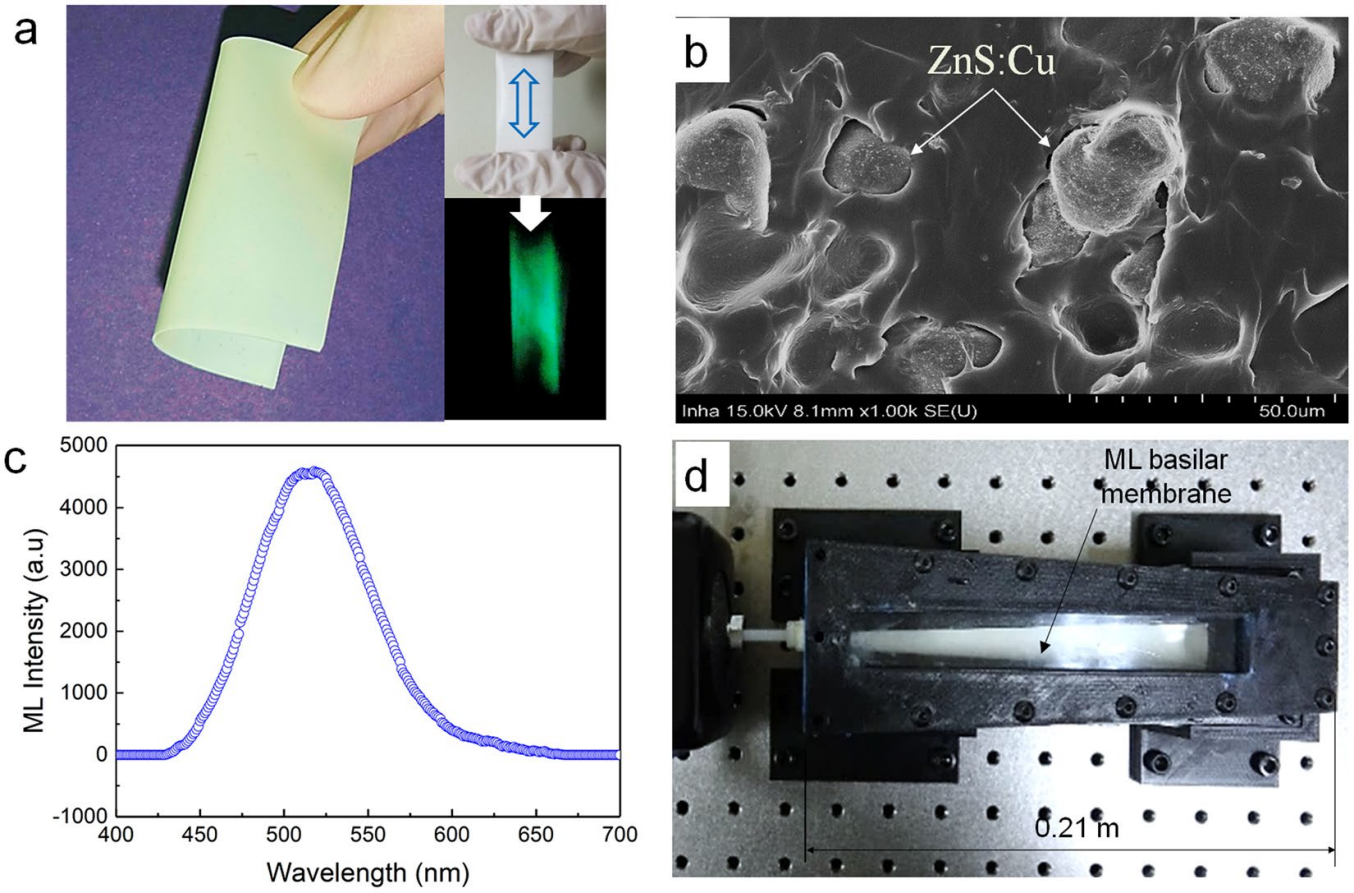
ZnS:Cu-polydimethylsiloxane (PDMS) composite used for the production of the ML basilar membrane. (**a**) Photographs and ML image under the cyclic load (5 N, 10 mm/s); (**b**) cross-sectional scanning electron microscopy image showing ZnS:Cu particle in PDMS matrix; (**c**) ML spectrum; (**d**) ML basilar membrane embedded inside artificial cochlea.

**Figure 5:**
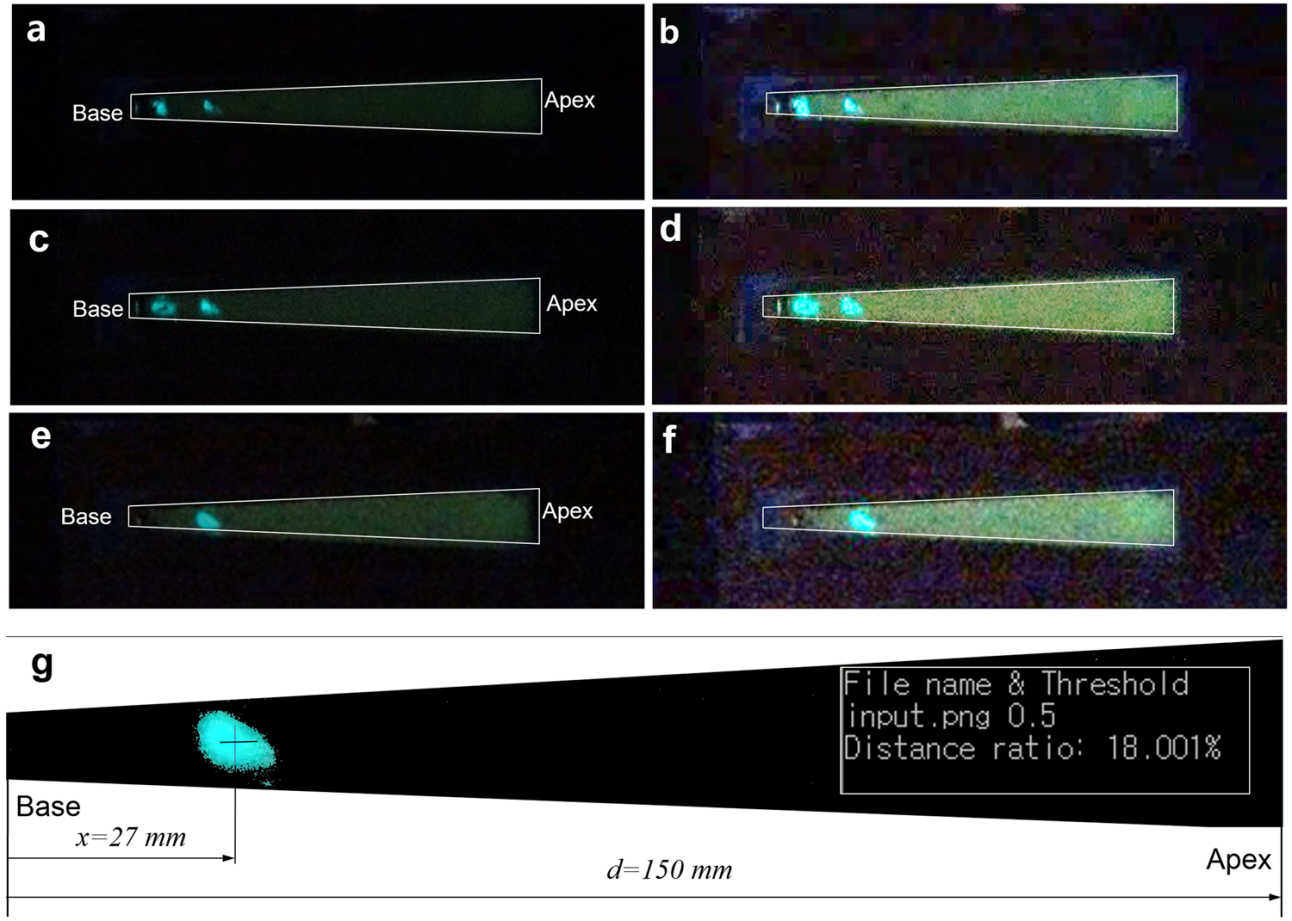
Typical examples of experimental result measured by digital camera (image sensor). (**a**,**b**) 110 Hz; (**c**,**d**) 80 Hz; (**e**,**f**) 40 Hz; (**g**) example of image processing using an image of (**e**) distance ratio: 18%; (**b**,**d**,**f**) are pseudo-colored images (brightness-filtered image to highlight the shape of BM).

**Figure 6:**
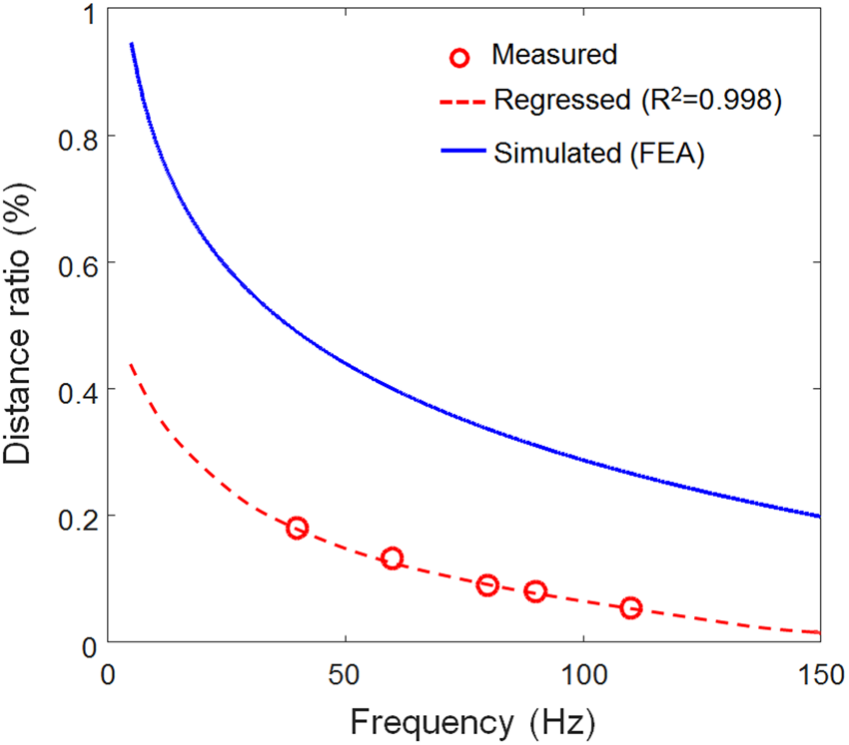
Comparison of sensitivity curves (distance ratio vs. frequency).

An ML basilar membrane ($t=0.6\,mm$) was first fabricated for initial prototype device, as shown in Fig. 4(a). As shown in the cross-sectional scanning electron microscopy (SEM) image shown in Fig. 4(b) and Figure S3, ZnS:Cu particles were uniformly mixed in polydimethylsiloxane (PDMS) matrix. The average particle size was calculated to be approximately 25 μm. Zooming at the interface of a ZnS:Cu particle and PDMS. The ML spectrum of the ML sheet measured by a spectrometer (DARSA PRO-5000 SYSTEM) is illustrated in Fig. 4(c). ML spectrum was measured upon stretching (strain rate of 10 mm/s) the ML sheet and produced peak emission at a wavelength of 518 nm (green emission). Although this peak emission wavelength of ZnS:Cu-PDMS composite can slightly vary depending on the stretching (strain) rate^34^, this blue-shift can be neglected because ML membrane does not vibrate in response to incoming frequency. Figure 4(d) shows the photograph of a frequency-selectivity prototype mimicking the human cochlea. Details of the overall experimental setup and additional information are described in Figure S4. Figure 5 shows the typical examples of images captured by a high-speed digital camera for the frequency of 110 Hz, 80 Hz, and 40 Hz, respectively. When the ML basilar membrane is strongly deformed by the travelling wave propagation associated with the position of maximal amplitude, ML-induced visible light can visualize the position of maximum amplitude. These positions (small area) could be quantified after image processing, as illustrated in Fig. 5(g). The camera exposure was intentionally set at 5 seconds owing to the low ML intensity of the fabricated ML membrane, although images could be captured in real time when the ML intensity was sufficient high for image processing. As a result, two peaks, as depicted in Fig. 5(a,c), were observed upon propagation of the traveling wave. The first peaks were used to determine distance ratios. The dominant B channel (green emission) of the input ML image was first extracted followed by calculation of the average distance from base. Details concerning this image processing are explained in the Methods section. The distance ratio, ratio of the distance (*x*) from base to the entire image length cropped from the original image (*d*) could then be measured. Distance ratios, thus measured, are depicted in Fig. 6, wherein sensitivity curves could be obtained through use of the following regressed (curve fitting) formula (dashed line).

1 $$y=-\,0.126\,\mathrm{ln}(x)+0.646$$

where *y* denotes the distance ratio and *x* denotes excitation frequency (Hz). The measured points seemed logarithmically proportional to the input frequency. However, it was difficult to capture ML light at low frequencies owing insufficient strain rates, in agreement with the fluid–structure coupled acoustic analysis shown in the velocities in response to harmonic excitation for different frequencies (Figure S1). This technical limitation could be addressed by improving the ML intensity via optimization of the mixing ratio^29^. The continuous distance ratio could be simulated using the coupled fluid–structure acoustic analysis (see Fig. 3) and compared as depicted in Fig. 6, wherein the sensitivity curve could be obtained by the following formula (blue solid line).

2 $$y=-\,0.22\,\mathrm{ln}(x)+1.31$$

**Figure 7:**
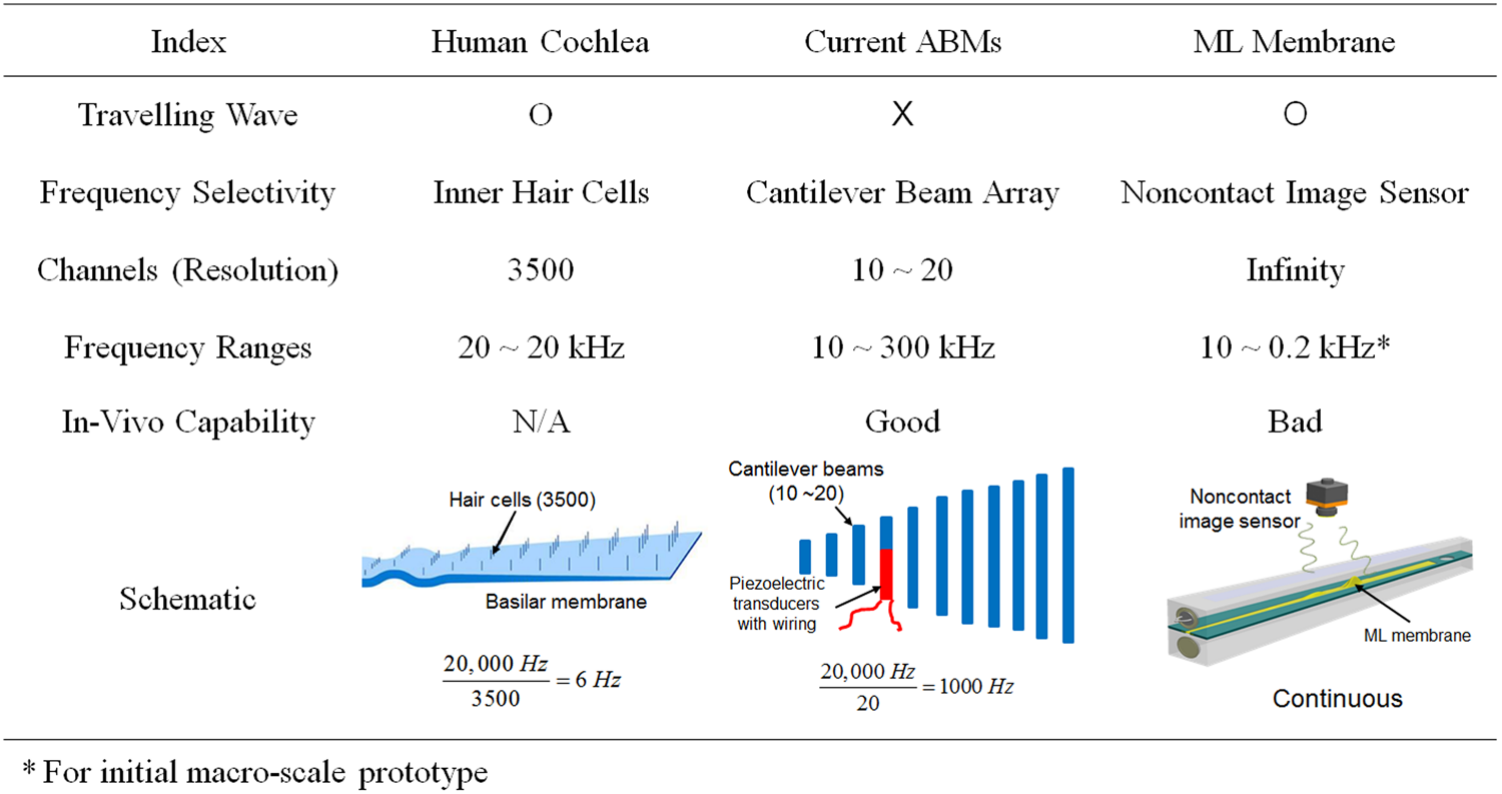
Comparison of three frequency selectivity approaches.

In fact, some difference exhibits between the measured and simulated sensitivity. The possible reason for this difference is that viscoelastic properties of ML membrane was assumed to be elastic, and ideal Rayleigh damping model was used for acoustic analysis. The sensitivity curve can be tuned by employing a different prototype design. For example, the position corresponding to 40 Hz could be moved along the apex direction (i.e., higher distance ratio) by using different viscous fluids (brake oil, typically higher effective bulk modulus than mineral oil) (Figure S5(b)) or an ML basilar membrane with a different thickness of 0.4 mm (original thickness: 0.6 mm), as depicted in Figure S5(c). Advantages and disadvantages of the proposed frequency-selectivity technique were compared against conventional MEMS type ABMs and have been summarized in Fig. 7. The frequency range of the ML membrane method is very small (10–150 Hz) compared to those of other techniques because the initial prototype used in this study comprised a macro scale test-bed (a few centimeter size) designed for initial assessment of the frequency selectivity method and aimed at simply mimicking the function of the human BM (a proof-of-concept study). Authors believe that the frequency ranges could be improved upon introduction of the MEMS technology at the micro scale.

## Conclusions

In this study, a new approach to achieve frequency selectivity for analyzing the frequency information of input wave (or sound) was experimentally explored. This approach attempts to mimic the function of the human BM within the cochlea using an ML membrane made of ZnS:Cu-PDMS composite. Using a fluid-structure coupled acoustic analysis (simulation) and an experiment with a prototype artificial cochlea with an ML membrane, we demonstrate that this new method achieves a frequency selectivity similar to that of the human cochlea, except for the width of frequency ranges. To the best of our knowledge, we report for the first time that it is possible to achieve a new means of determining the frequency information from the input sound (i.e., frequency selectivity) based on travelling-wave propagation on ML membrane. This method of using travelling wave for frequency selectivity appears to offer promising applications for acoustic sensory systems, although there are technical problems that remain and need to be further examined. Further studies will focus on the improvement of ML sensitivity, the extension of frequency ranges, and miniaturization of the prototype using MEMS technology.

## Methods

### Fabrication of ML Membrane

ML membrane was synthesized from the composite of commercially available ZnS:Cu (LONCO Company Limited) and PDMS (Sylgard 184 Silicone Elastomer). Liquid PDMS (Sylgard 184 A) with a curing agent (Sylgard 184 B) was initially added in a cylindrical plastic container at a weight ratio of 10:1, followed by 30 wt % of ZnS:Cu. In order to homogeneously disperse ZnS:Cu in PDMS and avoid agglomeration, a few 10-mm alumina grinding balls were added, and the container was transferred to a planetary shear mixer for 10 min at a mixing speed of 400 rpm. Finally, the composite was degassed inside a vacuum chamber for 10 min to remove entrapped air bubbles. In advance a 210 mm × 70 mm rectangular mold was prepared on a glass plate using a paper tape 0.30 mm thick. ZnS:Cu/PDMS composite was cast using the “doctor blade” technique. The glass plate was transferred to a 60 °C vacuum oven for 2 hours for the solidification. Solidified ML sheet was peeled off the glass plate and used for the experiments.

### Image processing of measured ML Image

The measured ML image was divided into R, G, and B channels. Since the B channel (green emission) of the input ML image was dominant, the threshold value of the B channel was then specified to extract the pixel value for the B channel. The pixel values in the R and G channels and below-threshold value in the B channel were set to zero. In order to calculate the average distance from the reference point (base) to non-zero pixels, we divided the number of non-zero pixels by the number of pixels between the non-zero pixels and the reference point. An Open CV (open source computer vision) library with the C++ interface was used for real-time image processing.

## Electronic supplementary material

Supplementary Information

Demonstration Movie

Demo video A

Demo video B
