# Supplementary material for: A Novel Frequency Selectivity Approach Based on Travelling Wave Propagation in Mechanoluminescence Basilar Membrane for Artificial Cochlea
Source: Sci Rep. 2018 Aug 13;8:12023. doi: 10.1038/s41598-018-30633-0 (PMC6089901; doi:10.1038/s41598-018-30633-0)
Supplement: Supplementary file 1 — Supplementary Information [file 41598_2018_30633_MOESM1_ESM.pdf]

Supplementary Information

A Novel Frequency Selectivity Approach Based on Travelling Wave Propagation in Mechanoluminescence Basilar Membrane for Artificial Cochlea

Yooil Kim[2], Ji-Sik Kim[3] and Gi-Woo Kim[1,*]

[1] *Department of Mechanical Engineering, Inha University, 22212, South Korea*

[2] *Department of Naval Architecture and Ocean Engineering, Inha University, 22212, South Korea*

[3]*School of Nano & Adv. Mater. Engineering, Kyungpook National University, 37224, South Korea*

*Address correspondence to Gi-Woo Kim, Department of Mechanical Engineering, Inha University, 100 Inha-ro, Nam-gu, Incheon, 22212, Republic of Korea. Tel: +82-32-860-7313; Fax: +82-32-868-1716;E-mail: gwkim@inha.ac.kr

Abstract

This study presents the initial assessment for a new approach to frequency selectivity aimed at mimicking the function of the basilar membrane within the human cochlea. The term cochlea tonotopy refers to the passive frequency selectivity and a transformation from the acoustic wave into a frequency signal assisted by the hair cells in the organ of Corti. While high-frequency sound waves vibrate near the base of the cochlea (near the oval windows), low-frequency waves vibrate near the apex (at the maximum distance from the base), which suggests the existence of continuous frequency selectivity. Over the past few decades, frequency selectivity using artificial membranes has been utilized in acoustic transducers by mimicking cochlea tonotopy using cantilever-beam arrays with defined physical parameters such as

length and thickness. Unlike the conventional cantilever-beam array type, the travelling wave propagation based-mechanoluminescence (ML) membrane made of ZnS:Cu- polydimethylsiloxane (ZnS:Cu-PDMS) composite that we describe here provides frequency selectivity more similar to that demonstrated by the human membrane. Here, we explored the potential of the ML membrane to deliver new frequency selectivity by using a non-contact image sensor to measure the visualized frequencies. We report that the ML basilar membrane can provide effective visualization of the distribution of strain rate associated with the position of maximal amplitude of the travelling wave.

Keywords: Artificial Cochlea, Basilar Membrane, Frequency Selectivity, Fluid-structure Coupled Acoustic Analysis, Travelling Wave Propagation, Mechanoluminescence (ML), ZnS:Cu-PDMS Composite

Supplementary #1: Fluid-structure Interaction Acoustic Analysis of Human Basilar Membrane

The travelling wave patterns of human basilar membrane for different frequencies are illustrated in Figure S1. The different position of peak is proportional to the incoming frequency, which implies that human BM exhibit the frequency selectivity over the frequency range between 20 Hz and 20 kHz. As increased the frequency, these peak points moved forward into the base of BM [1].

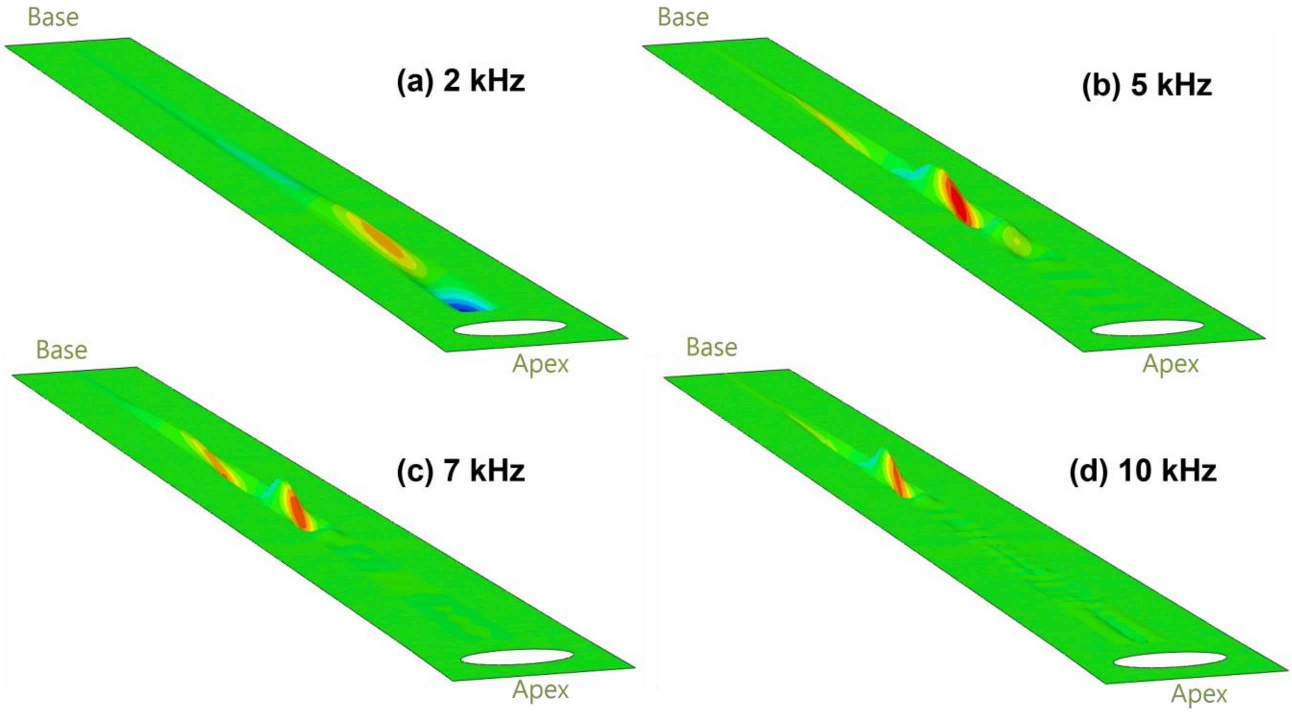

Figure S1. Travelling wave patterns for different frequency, indicating a different location of peak. (a) 2 kHz, (b) 5 kHz, (c) 7 kHz, and (d) 10 kHz

Supplementary #2: Fluid-structure Interaction Acoustic Analysis of ML basilar Membrane

Figure S2 shows the velocity of ML basilar membrane in response to a harmonic velocity input of OW for different frequencies. However, the velocity of ML BM at the low frequency such as 25 Hz is relatively smaller compared to the velocity of ML membrane at the high frequency.

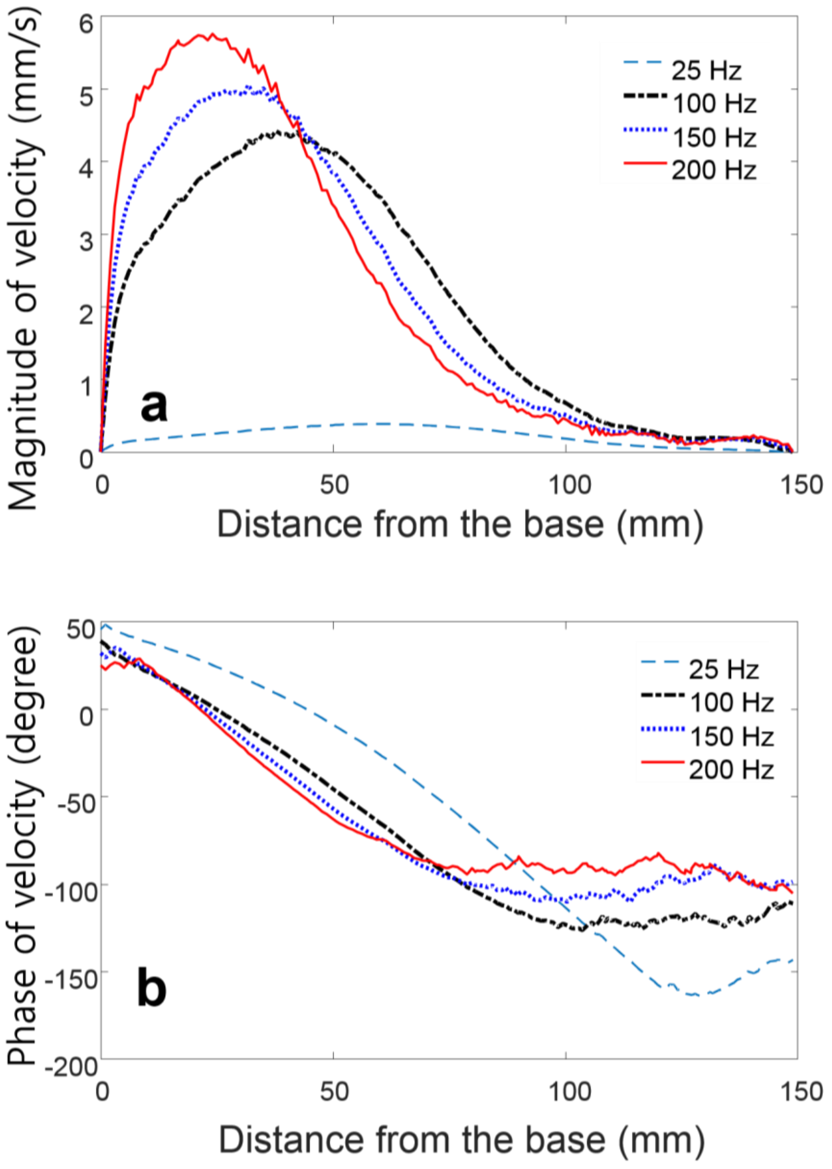

Figure S2. The velocity of ML BM in response to harmonic excitation (velocity) for different frequencies. (a) magnitude (b) phase

Supplementary #3: Additional SEM image of ML membrane

Figure S3 shows the additional SEM images to show the ZnS:Cu particle dispersion within PDMS matrix.

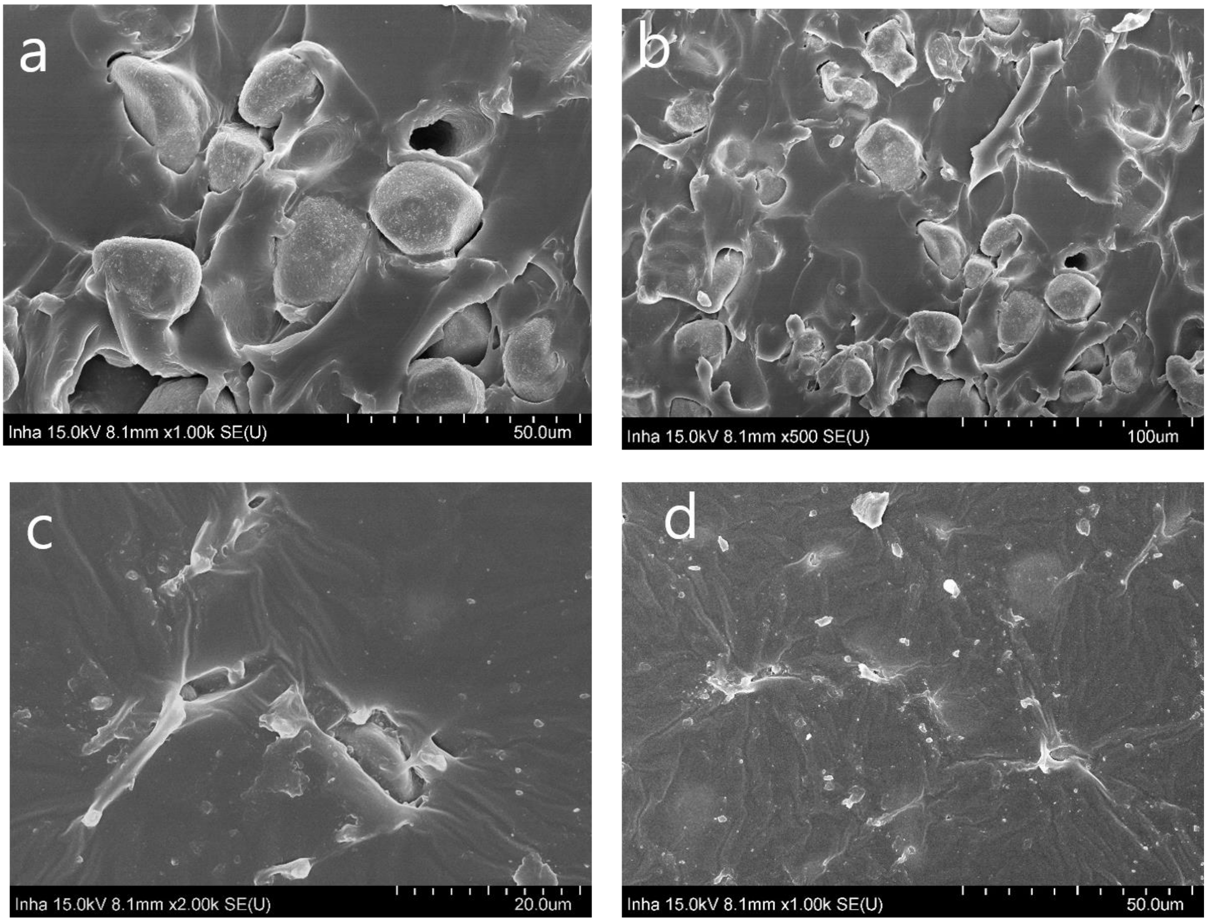

Figure S3. Scanning electron microscopy (SEM) image indicating ZnS:Cu particle dispersion within PDMS matrix; (a) cross section, x1000, (b) cross section, x500, (c) surface, x2000, and (d) surface, x1000.

Supplementary #4: Experimental Set-up

Figure S4 shows overall experimental set-up for measuring the ML emission occurring in the position where the travelling wave propagation reached its maximum amplitude. A digital high-speed camera was used as an image sensor to detect the position where the vibrating ML membrane exhibits the maximum amplitude by capturing the ML light emission via transparent glass. The camera exposure time (i. e., shutter speed) was set to be 5 second to increase the amount of light that can reach the image sensor. A compact electromagnetic shaker (K2004E01 SmartShaker, The Modal Shop) integrated with a power amplifier was used to harmonically excite the OW. The sinusoidal input signal with different frequencies was generated by using a waveform generator (Agilent 33522A), and applied to the shaker. An experiment is normally performed when the laboratory room can be made completely dark (e.g., at night) because the ML phenomenon needs to be operated in complete darkness to improve the ML sensitivity. This technical limitation could be fully overcome considering the stand-alone artificial cochlea where an image sensor is embedded inside of artificial cochlea, and it must be completely dark.

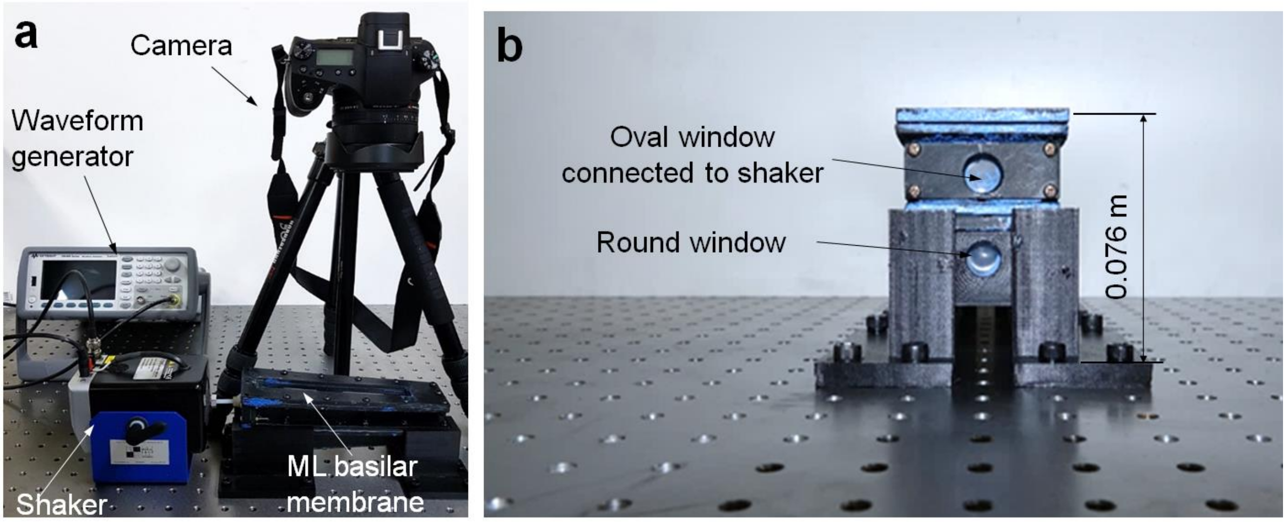

Figure S4. Overall experimental set-up. a) Photograph b) front view showing two windows

Supplementary #5: Additional Experiment Results

Figure S5 shows the tuning of frequency selectivity using different design parameters. For example, the position corresponding to 40 Hz can be changed by using different viscous fluid (brake oil), as shown in Figure S5 b. This position also can be tuned by using a ML basilar membrane with different thickness of 0.4 mm (original: 0.6 mm), as shown in Figure S5 c.

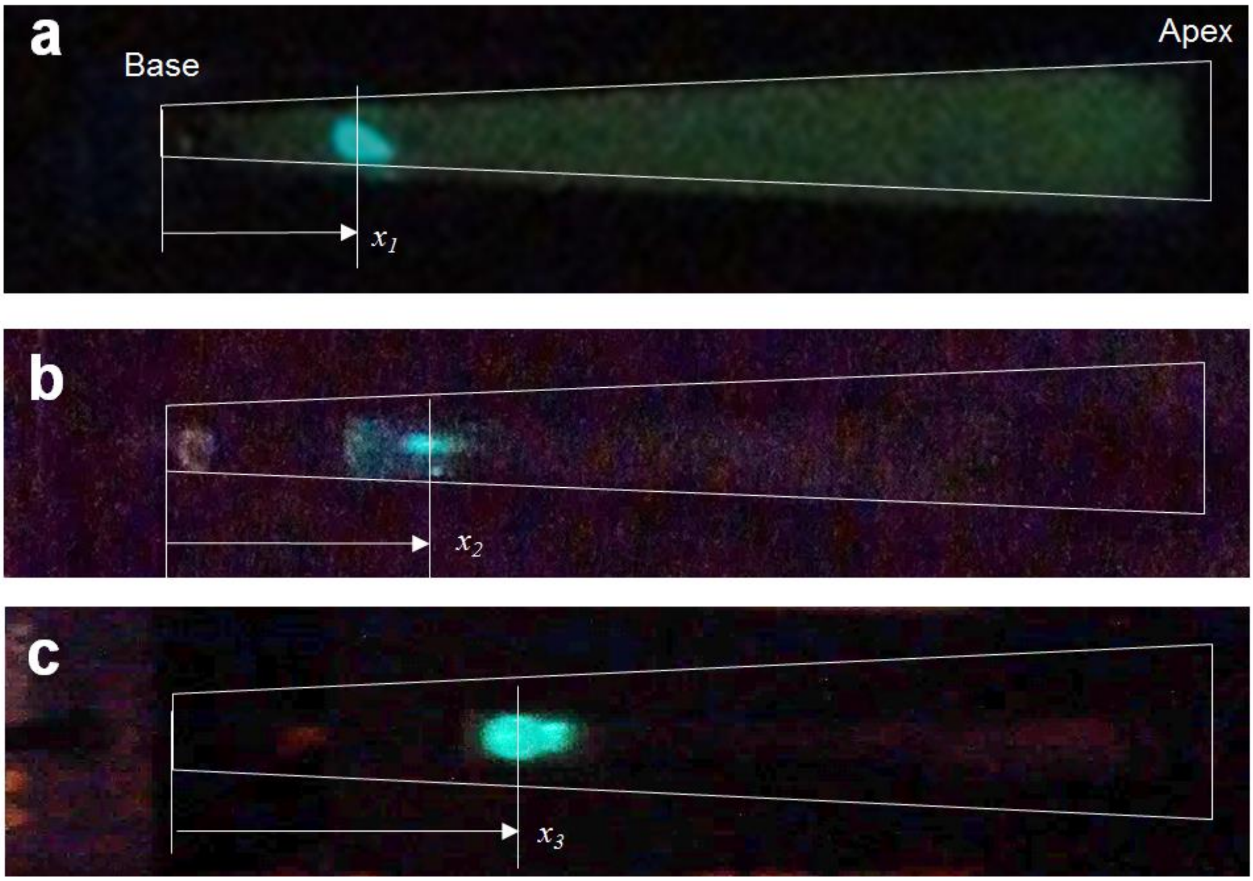

Figure S5. Experimental results. (a) original AM model (b) with different oil (brake oil), (c) with different thickness of ML basilar membrane at the frequency of 40 Hz.

Additional Information

Supplementary information accompanies this paper with demo movie and GIF images that animate travelling wave patterns for different frequency, indicating a different location of peak frequency.

References

1. Yooil Kim, Jeong-Hwan Kim, and Gi-Woo Kim, *Proc. SPIE Vol. 10598, Sensors and Smart Structures Technologies for Civil, Mechanical, and Aerospace Systems*, **10598-62**, (2018)
